# Development and Validation of Specific Carotene Food Composition Tables for Use in Nutritional Epidemiologic Studies for Japanese Populations

**DOI:** 10.2188/jea.11.266

**Published:** 2007-11-30

**Authors:** Yoshiko Takahashi, Satoshi Sasaki, Shoichiro Tsugane

**Affiliations:** 1 Epidemiology and Biostatistics Division, National Cancer Center Research Institute East, Kashiwa, Japan.; 2 Department of Environmental and Occupational Health, Toho University School of Medicine, Tokyo, Japan.

**Keywords:** dietary assessment, epidemiology, nutrition, carotene

## Abstract

Assessment of dietary intake is important to understand the relationship between nutrition and health. Although the role of specific carotenoids has recently been of great interest, there are no comprehensive food composition tables for intake of specific carotenoids in Japan. We have therefore developed a new carotene food composition table that shows the alpha- and beta-carotene values based on an extensive review of the literature (FCT1). Using a 14- or 28-day diet record data of sample population (n=188), we selected 12 important foods to two carotene intakes. We analyzed the carotene contents of the foods, and developed the another composition table in which the food contents were replaced by the analytical values (FCT2). Carotene intakes of the population were significantly different between these two composition tables. However, the correlations between the dietary intake and the serum concentrations were almost identical, i.e., partial correlations using FCT1/FCT2 were 0.32/0.30 and 0.33/0.36 for alpha-carotene and 0.28/0.28 and 0.30/0.29 for beta-carotene in men and women. The similar correlations with the serum concentrations may indicate an comparable value for ranking individuals between the two tables. However, the results were inconclusive for the estimation of absolute intakes.

## INTRODUCTION

Assessment of dietary intake is important to understand of the relationship between nutrition and health by epidemiological investigation. Dietary assessment depends on the quality of the food composition tables used for nutrient calculation. Japanese food composition tables, however, do not always provide sufficient information such as sampling methods, detailed analytic methods and explanations of missing values.

In Western countries, although findings have not been consistent, several recent epidemiologic studies have reported the relationship between specific carotenoids in foods and the incidence of cancer at several sites^1^^-^^6^^)^ cardiovascular disease^7^^)^, and age-related macular degeneration^8^^)^. In Japan, however, there is no report to date of a comprehensive food composition table of specific carotenoids.

In this article, we describe the development of a new carotene food composition table that indicates the values for alpha- and beta-carotene. Firstly, we developed a carotene food composition table using published data on carotene food composition. Secondly, we sampled and analyzed the compositions of foods with high contribution to carotene intakes. Thirdly, the analyzed values were replaced by the literature-based values. Lastly, they were applied to a sample population, and validated by diet-serum relationships.

## METHODS

### 1. Development of new composition table using published data

We extensively searched the English and Japanese literature for reports on the food composition of specific carotenoids. We especially checked all the articles published in the following Japanese journals between 1990 and 1999 using “carotene or carotenoid, and several food names” in the title as key words: “J Food Sci Tec”, “J Jpn Soc Nutr Food Sci”, “Jpn J Nutr”, “Bull Jpn Soc Sci Fish” and “Nippon Nogeikagaku kaishi”. We also collected food composition tables which was used in the published epidemiologic studies on the association between carotene intake and diseases conducted in Western countries. We obtained 6 articles published in Japan^9^^-^^14^^)^, 3 in Finland^15^^-^^17^^)^, and the United States’ Department of Agriculture (USDA) food composition tables^18^^)^. The reports published in Japan covered only alpha- and beta-carotene, and no other carotenoids (beta-cryptoxanthin, lutein, zeaxanthin and lycopene) were mentioned. Thus, we developed a composition table only for alpha- and beta-carotene. Gamma-carotene and delta-carotene are determined in only a few vegetables^15^^)^. Furthermore, there are no reports about these carotenes neither in Japan nor in Western countries. Thus, we could not developed food composition tables of these carotenes. According to the literature reviews, the composition ratio of alpha- and beta-carotene was relatively stable, although the analytical values varied widely. The values of “carotene” in the standard food composition table for Japanese foods were almost the sum of the alpha- and beta-carotenes (K. Odaka, personal communication). Therefore, we developed a new carotene composition table by substituting the composition ratios of alpha- and beta-carotene obtained from the literature. Since our new carotene composition table is intended for Japanese populations, we have given priority to the reports published in Japan for the selection. The alpha- and beta-carotene composition of food was determined using the total carotene composition in the standard food composition table of Japanese foods (fourth revised edition)^19^^)^ and the ratio of alpha- and beta-carotene composition of the food obtained from the literature as follows (Method A):

$$C=ST*(R_{\text{i}}/R_{\text{t}})\;\text{(}\mu \text{g/100 g food portion)},$$ (1)

where C is the estimated specific carotene content of a food, ST is the total carotene content in the standard food composition table of the food, R_i_ is the specific carotene content of the food obtained from the literature, i is alpha-carotene or beta-carotene, and R_t_ is the total carotene (sum of alpha- and beta-carotene) content obtained from the literature.

When specific carotene data were unavailable for a particular food, we used the composition ratio of alpha- and beta-carotene of the botanically similar foods with known specific carotene content (Method B). In reports published in Japan, most of the data were analyzed using raw foods. Thus, the carotene content ratios of raw foods were adapted to that of cooked foods. We estimated specific carotene compositions for foods with carotene contents >0 *μ*g/100 g portion. Foods categorized as cooked and prepared foods, the so-called “food group 18” in the standard food composition table, were not included in this study. Among confectioneries, contained at least some carotene, mostly from milk, egg, butter, and color additives. Based on our literature search, these ingredients contain only beta-carotene. Total carotene was thought to be beta-carotene in this food group (Method C). Although green leafy vegetables were considered carotene-rich foods, we could obtain data only for a part of these foods. Most green leafy vegetables do not contain alpha-carotene according to the published reports. Total carotene was thought to be beta-carotene in green leafy vegetables (Method C). Algae are also carotene-rich foods, but reliable information on the alpha- and beta-carotene composition was unavailable. “Nuts and seeds” and most “seasonings and spices” also contain carotene, but we could not obtain their specific carotene composition.

### 2. Selection and laboratory analysis of important foods

Carotene compositions were analyzed for the important foods selected by the following criteria. First, specific carotene intake levels of the four sample populations (see “sample population and data collection” in detail) were calculated with the developed carotene composition table. Next, we selected foods in which alpha- or beta-carotene contributed more than 3% of the overall intake in each area. The food samples were purchased between June and July in 1999. Three samples were obtained for each item from 3 different stores. Most samples were from local markets, and the cheapest one was selected.

Only the edible part of the foods was used for laboratory analysis. Fifty grams of each sample, 50 mL of water, and 5 mL of pyrogallol were mixed together. Ten grams of samples were homogenized and extracted in an Ultra Turrax by 150 mL of n-hexane / acetone (2:1), then filtered and the food residue extracted several times with n-hexane / acetone until the yellow became colorless. The combined carotene extract was mixed with 100 mL of water for 30 min at room temperature. After phase separation, the lower phase was removed and shaken again with hexane. The recombined n-hexane phases were dried by water-free Na_2_SO_4_ and reduced in the rotation evaporator at 30°C. Then 25 *μ*L of the solution was applied to a high-performance liquid chromatography (HPLC) (Waters, Milford, Mass). The HPLC conditions were as follows: C18 reverse phase column, Wakopak (Wako, Osaka, Japan); mobile phase, methanol: acetonitrile: water=60:40:1; UV wavelength, 450; flow rate, 1.0 mL/min. The mean value was calculated with the data from triplicate analysis.

### 3. Development of table with analytical (FCT2)

We computed alpha- and beta-carotene composition using the formula (1) for the foods with analytical values. But R_i_ is the analytical specific carotene content of the food and R_t_ is the sum of analytical alpha- and beta-carotene content of the food (Method D). Botanically similar foods were also replaced by analytical values (Method E). The composition for these foods in FCT1 were replaced by the new compositions. We referred to this composition table as FCT2.

### 4. Sample population and data collection

#### Subjects

The sample population was a sub-population (n=221, aged 44-63 years) from the Japanese Public Health Center-based Prospective Study (JPHC Study)^20^^)^ living in Iwate, Akita, Nagano, and Okinawa prefectures who kept 28- or 14-day dietary records (DR) in 1994 or 1995. The subjects were orally informed the purpose and procedure of this study and agreed to the participation by providing diet record and blood sample.

#### Diet record

Semi-weighted dietary records from four different seasons (two seasons, i.e., winter and summer only in Okinawa) over seven consecutive days were collected by a method used in the National Nutrition Survey^21^^)^ with some modifications^22^^, ^^23^^)^. Research dieticians instructed the subjects to record all foods and beverages prepared and consumed in a specially designed booklet. The participants were asked to provide detailed descriptions of each food, including the methods of preparation and recipes whenever possible. The dieticians checked the records at each participant’s home during the survey and reviewed them in a standardized way after recording.

#### Blood samples

A total of 25 mL serum was collected by venipuncture from all subjects just before the DR in winter and just after the DR in summer. Fasting for at least 5 hours was requested before blood collection. The samples were stored at -80°C until analysis. Serum carotenes were determined by HPLC. Total cholesterol in serum was analyzed enzymatically with an autoanalyzer.

#### Anthropometric data

The body mass index (BMI) was computed in all subjects as self-reported weight (kg) divided by height (m) squared.

### 5. Validation of two composition tables

We calculated specific carotene levels of the sample population with the two developed carotene composition tables. The first step was to compare the distributions of each of the aforementioned composition tables. We evaluated the agreement between the two different composition table estimates by comparing mean and median intakes. Because the distributions of the paired differences were not normally distributed, a nonparametric test, the Wilcoxon signed-rank test, was used to determine whether or not the median difference between FCT1 and FCT2 carotene estimates were significantly different from zero. The second step was to validate the two composition tables by comparing the diet-serum relationship for the alpha- and beta-carotene using Spearman rank correlation coefficients. Multiple regression analysis was conducted on the serum carotene concentrations and the estimated intakes including possible confounding factors such as BMI, serum cholesterol concentration, alcohol intake, number of cigarettes smoked per day, and age^24^^-^^27^^)^. Serum total- and beta-carotene showed significant correlations with BMI and alcohol intake. Alpha-carotene showed significant correlations with BMI, alcohol intake, and serum total cholesterol. Therefore the partial correlation coefficients adjusting for these significant variables were computed in each model and presented here. SAS software, release 6.12 (SAS Institute, Cary NC) was used for all statistical computations.

Three subjects (1 man and 2 women) who took carotene containing supplements were excluded from the analysis. Serum carotene reflects the relatively long-term intake such as several weeks^28^^, ^^29^^)^. In addition, there are large day-to-day variation in dietary carotenoid intake^30^^-^^32^^)^. Thus, we used serum measurement in summer and 14-day DR data combining two 7-day DR data obtained in winter and summer respectively.

## RESULTS

### 1. Development of composition table using published data

Table 1 shows the number of foods by substitution methods. Among 553 foods in which carotene is >0 *μ*g/100 g portion, substitution methods were found for 341 foods. A suitable substitution method was not found for the remaining 212 foods. The 81.5%, 11.1%, and 7.3% of foods were substituted by Methods A, B and C, respectively. The food compositions of alpha- and beta-carotenes for the 341 foods are listed in Appendix A. Appendix B shows there was only beta-carotene.

**Table 1.** The number of foods by substitution method and food group ^a, b^.

	Food with carotene (>0 µg/100 g portion )								Foods without carotene (µg/100 g portion=0)	Total

	Substituted						Not substituted	Total		

	Based on literature value			Based on analytical value		Total				
	
	Method A	Method B	Method C	Method D	Method E					
Cereals	5	1	0	0	0	6	0	6	128	134
Potatoes and starches	4	0	0	0	0	4	2	6	28	34
Sugar and sweeteners	0	0	0	0	0	0	0	0	25	25
Confectioneries	2	0	13	0	0	15	3	18	96	114
Fats and oils	0	0	0	0	0	0	0	0	11	11
Nuts and seeds	0	0	0	0	0	0	29	29	6	35
Pulses	13	0	0	0	0	13	9	22	39	61
Fishes and shellfishes	0	0	1	0	0	1	37	38	295	333
Meats	0	0	0	0	0	0	6	6	201	207
Eggs	13	4	0	0	0	17	0	17	3	20
Milks	35	0	0	0	0	35	0	35	15	50
Vegetables	134 (107)	18 (14)	11 (11)	0 (27)	0 (4)	163 (163)	43	206	49	255
Green & yellow vegetables	87 (62)	11 (11)	8 (8)	0 (25)	0 (0)	106 (106)	2	108	1	109
Others	47 (45)	7 (3)	3 (3)	0 (2)	0 (4)	57 (57)	41	98	48	146
Fruits	59 (58)	15 (15)	0 (0)	0 (1)	0 (0)	74 (74)	15	89	44	133
Fungi	0	0	0	0	0	0	0	0	31	31
Algae	0	0	0	0	0	0	40	40	4	44
Beverages	10	0	0	0	0	10	2	12	53	65
Seasonings and spices	3	0	0	0	0	3	26	29	26	55
Total	278 (250)	38 (34)	25 (25)	0 (28)	0 (4)	341 (341)	212	553	1054	1607

^a^ Refer to substitution methods.

^b^ Values indicate the number of foods by literature-based substitution (FCT1) and the values in parentheses indicate the number of foods after analytical values were included (FCT2).

### 2. Selection and analysis of important foods

Table 2 presents the contribution rate of food for intake of total-, alpha-, and beta-carotene in the sample population. Ninjin (*Daucus carota*) was the most important food both for alpha- and beta-carotene intakes in all areas (about 90% for alpha-carotene, and 32 to 50% for beta-carotene). No apparent area-difference was observed for alpha-carotene except in the Okinawas, where Nigauri (*Momordica charantia*) was also important for alpha-carotene intake. In all the areas, hourensou (*Spinacia oleracea*) mainly contributed to beta-carotene intake. In the Nagano area, Nozawana (*Brassica campestris*) also largely contributed to beta-carotene intake. In the Okinawa area, mango (*Mangifera indica*) also acconuted for much beta-carotene intake. According to the criteria, we selected 12 foods and collected 72 samples in each area. Ninjin and hourensou were collected in 4 areas. Nira (*Allium tuberosum*) and tomato (*Lycopersicon esculentum*) were collected in the Iwate, Akita, and Nagano areas. Komatsuna (*Brassica campestris*) was collected in the Akita and Nagano areas. Shungiku (*Chrysanthemum coronarium*) was gathered only in the Akita area. Seiyou-Kabocha (*Cucurbita mexima*) was obtained from the Iwate and Nagano areas. Nozawana and Ingenmame (*Phaseolus vulgaris*) were collected only in the Nagano area Karashina (*Brassica juncea*), Nigauri, and mango were gathered only in the Okinawa area.

**Table 2.** Contribution of foods^a^ to alpha- and beta-carotene intakes by area based on 14-day dietary record for 88 men and 100 women using a literature-based food composition table (FCT1).

Iwate (n=47)		Akita (n=50)		Nagano (n=44)		Okinawa (n=47)		Total (n= 188)	
				
Food	Cumulative %	Food	Cumulative %	Food	Cumulative %	Food	Cumulative %	Food	Cumulative %
Alpha-carotene									
Ninjin, raw	92.6	Ninjin. raw	91.9	Ninjin, raw	90.4	Ninjin, raw	94.0	Ninjin, raw	92.3
Tomato, raw	96.0	Tomato, raw	95.9	Tomato, raw	94.3	Nigauri, raw	97.3	Tomato, raw	95.7
------		------		Ingenmame, raw	97.4	------		------	
Beta-carotene									
Ninjin. raw	46.5	Ninjin. raw	33.1	Ninjin, raw	32.2	Ninjin, raw	49.9	Ninjin, raw	39.9
Hourensou. raw	62.2	Hourensou, raw	53.0	Nozawana, salted	49.3	Hourensou, raw	61.8	Hourensou, raw	54.2
Nira, raw	67.3	Nira, raw	59.1	Hourensou, raw	58.3	Mango	66.3	Nozawana, salted	59.0
Seiyou-Kabocha. raw	70.4	Komatsuna, raw	65.1	Komatsuna, raw	65.1	Karashina, raw	70.3	Nira, raw	63.7
------		Shungiku, raw	68.7	Nira, raw	69.7	------		Komatsuna, raw	67.9
------		------		Seiyou-Kabocha, raw	73.7	------		Seiyou-Kabocha, raw	71.0
------		------		Tomato, raw	76.9	------		------	
Total carotene									
Ninjin, raw	50.6	Ninjin, raw	38.2	Ninjin, raw	37.2	Ninjin, raw	56.7	Ninjin, raw	45.2
Hourensou, raw	62.4	Hourensou, raw	54.0	Nozawana, salted	50.9	Hourensou, raw	66.0	Hourensou, raw	56.3
Nira, raw	66.2	Nira. raw	20.7	Hourensou, raw	58.1	Mango	69.5	Nozawana, salted	60.0
------		Komatsuna, raw	63.6	Komatsuna, raw	63.5	Karashina, raw	72.6	Nira, raw	63.7
------		------		Nira, raw	67.2	------		Komatsuna, raw	67.0
------		------		Tomato, raw	70.5	------		------	
------		------		Seiyou-Kabocha, raw	73.7	------		------	

^a^ Ninjin= *Daucus carota*; Tomato= *Lycopersicon esculentum*; Hourensou= *Spinacia oleracea*; Nira= *Allium tuberosum*;

Seiyou-Kabocha= *Cucurbita mexima*; Komatsuna= *Brassica campestris*; Shungiku= *Chrysanthemum coronarium*;

Ingenmame= *Phaseolus vulgaris*; Nozawana= *Brassica campestris*; Nigauri= *Momordica charantia*;

Mango= *Mangifera indica*; Karashina= *Brassica juncea* (scientific names are in Italics).

Analytical contents of alpha- and beta-carotene were shown in Table 3. Although slight differences were found for the contents between areas, there were no apparent differences in the content ratio of alpha- and beta-carotene between samples.

**Table 3.** Analytical contents of alpha- and beta-carotene (µg/100 g food portion).

	Number of samples	Alpha-carotene			Beta-carotene			Total-carotene		Total-carotene in the standard tables of food composition table, the fourth revised edition
		
		Mean	( Range )	Ratio^b^	Mean	( Range )	Ratio^b^	Mean	( Range )	
Ninjin^c^	12	3525	(2774-5415)	0.26	9963	(7102-12751)	0.74	13488	(9827-18166)	7300
Hourensou^c^	12	---^a^		---	3455	(2628-4458)	1.00	3455	(2628-4458)	5200
Nira^c^	9	---^a^		---	2895	(1817-4409)	1.00	2895	(1817-4409)	3300
Tomato^c^	9	306	(247-369)	0.28	791	(625-966)	0.72	1097	(872-1287)	390
Seiyou-Kabocha^c^	6	---^a^		---	4733	(3595-6546)	1.00	4733	(3395-6546)	850
Komatsuna^c^	6	---^a^		---	2081	(1890-2383)	1.00	2081	(1890-2383)	3300
Ingenmame^c^	3	156	(144-166)	0.24	492	(441-535)	0.76	649	(592-690)	480
Shungiku^c^	3	---^a^		---	2164	(1756-2538)	1.00	2164	(1756-2538)	3400
Nozawana^c^, salted	3	---^a^		---	939	(908-993)	1.00	939	(908-993)	2100
Karashina^c^	3	744	(719-772)	0.35	1387	(1365-1405)	0.65	2130	(2105-2177)	2300
Nigauri^c^	3	202	(199-205)	0.30	462	(446-472)	0.70	664	(377- 645)	250
Mango^c^	3	159	(157-160)	0.15	872	(844-900)	0.85	1031	(1001-1060)	1600

^a^ Not detected.

^b^ Composition ratio of alpha- or beta-carotene to total carotene.

^c^ See Table 2 for detailed information.

The number of foods in the revised composition table by the substitution method was shown in Table 1 (in parentheses). Carotene contents were revised with analytical values for 32 foods (Methods D and E).

### 3. Validation of two composition tables

Mean, standard deviation, and median estimates of daily carotene intakes based on the developed composition tables in the sample populations by sex are shown in Table 4. The estimated alpha-carotene intakes were higher in FCT1 than in FCT2. Conversely, the estimated beta-carotene intakes were lower in FCT1 than in FCT2. All the median values are significantly different between FCT1 and FCT2 by Wilcoxon signed-rank test. We also calculated and examined them by area, but no apparent area-difference was observed (data not shown). Table 5 shows Spearman correlation coefficients for specific carotenes between the intakes and the corresponding serum concentrations. In both sexes, carotene intakes moderately correlated with the serum concentrations. Only small differences were found between the results based on FCT1 and FCT2.

**Table 4.** Daily carotene intakes (µg) estimated using developed carotene food composition tables^a^.

	FCT1			FCT2			Statistical differences^b^	Correlation^d^
	
	Mean (SD)		Median	Mean (SD)		Median		
Men (n=88)								
Total-carotene	3420	(1506)	2969	-----^c^				
Alpha-carotene	555	(327)	437	497	(303)	385	p<0.001	0.99
Beta-carotene	2670	(1217)	2359	2728	(1222)	2416	p<0.001	0.99

Women (n=100)								
Total-carotene	3347	(1392)	3160	-----^c^				
Alpha-carotene	515	(269)	456	462	(238)	394	p<0.001	0.97
Beta-carotene	2644	(1149)	2492	2697	(1160)	2522	p<0.001	0.99

^a^ SD=standard deviation; FCT1= literature-based table; FCT2= table developed by combining literature and analytical values (see text for detail).

^b^ Statistical difference of median between FCT1 and FCT2 by Wilcoxon signed-rank test.

^c^ Same as FCT1 (see text for details).

^d^ Spearman rank correlation coefficient between FCT1 and FCT2 estimates.

**Table 5.** Spearman rank correlation coefficients between carotene intakes (µg/day) and corresponding serum concentrations (mg/mL) in a sample population.

	Men			Women		
	
	FCT1^a^		FCT2^a^	FCT1^a^		FCT2^a^
Simple correlations		n=88			n=100	
Total carotene	0.28		---^b^	0.25		---^b^
Alpha-carotene	0.35		0.34	0.36		0.38
Beta-carotene	0.27		0.26	0.24		0.24
Partial correlations^c^		n=84			n=97	
Total carotene	0.28		---^b^	0.30		---^b^
Alpha-carotene	0.32		0.30	0.33		0.36
Beta-carotene	0.28		0.28	0.30		0.29

^a^ See Table 4 for the detailed definitions.

^b^ Same as FCT1 (see text for details).

^c^ Partial correlation coefficients adjusted for body mass index (BMI) and alcohol intake for total- and beta-carotene, and BMI, alcohol intake, and serum total cholesterol for alpha-carotene.

## DISCUSSION

Several study groups have developed food composition tables for use in epidemiologic studies by several methods^15^^, ^^33^^-^^37^^, ^^39^^)^, and they have often been evaluated for reliability^33^^, ^^35^^, ^^36^^, ^^39^^-^^43^^)^. Recent interest in the potential health effects of several carotenoids other than their contribution to vitamin A activity has stimulated the development of an accurate composition table for carotenoids in the United States^35^^, ^^36^^)^, Finland^15^^-^^17^^)^, and other countries.

Although our primary purpose in this study was to develop a carotenoid composition table for Japan, our composition tables were limited only to alpha- and beta-carotenes due to the lack of reliable information on other carotenoids such as beta-cryptoxanthin, lutein, zeaxanthin, and lycopene. Moreover, the reports published in Japan examined only alpha- and beta-carotene compositions. Although the literature in other countries referred to other carotenoids, information on original Japanese vegetables such as komatsuna, and nozawana was insufficient. Furthermore, because of the very limited nature of the reports published in Japan, in selecting these reports we could not sufficiently consider the data quality of the measuring and sampling methods used in them. Mangels et al. developed a carotenoid composition table for American foods using reference values obtained from more than 180 articles published in the US and other countries^36^^)^. Further literature research with larger sample size and reasonable quality is needed to obtain adequate food composition data on carotenoids in Japan.

Total carotene, alpha- and beta-carotene were mainly derived from Ninjin. Total-carotene and beta-carotene were derived from Hourensou and Nozawana, and alpha-carotene was derived from Tomato. Ninjin and Hourensou were also major sources of total carotene intake in two previous studies in Japan^44^^, ^^45^^)^.

Both analytical and reference values varied widely between sampled foods because many factors such as varietal differences, variable growth and storage, different geographic locations, and seasons, affect the carotene composition in foods^9^^, ^^36^^)^. This may seriously distort the results, especially in the development of a composition table using reference values rather than analytical values obtained from the foods actually consumed by a target population. Despite this assumption, the two composition tables similarly ranked the sample subjects according to each carotene intake when the serum carotene concentration was used as the gold standard (Table 5). The result indicates that analysis of foods consumed by a target population, at least with the method used in this study, may not improve results for ranking individuals by intakes. Several studies reported correlations for carotenes between the intakes assessed with DR and the serum or plasma concentrations in Western populations^27^^, ^^46^^)^. The correlations observed in the present study were comparable to or slightly lower than the previously reported values. But FCT1, which was developed using only reference values, may be more useful in nutritional epidemiologic studies conducted in various populations in Japan, because the analytical values of foods used in this study may not be considered representative. Moreover, we could not examine which composition table was preferable for use in the study to estimate “absolute” intake levels in the absence of a gold standard.

In conclusion, we developed a carotene composition table with food compositions obtained from the literature and analytical values for use in nutritional epidemiologic studies for Japanese populations. FCT1 is readily available for non-profit use only on the authors’ website (http://www.east.ncc.go.jp/epi). They request, however that this article be cited when a study in which the data, even in part, have been published or made available to the public. The composition table may be useful for studies on the association between alpha- and beta-carotene intakes and health status in Japanese populations. Its reliability for estimation of estimate absolute intakes, however, is not yet confirmed.[Table tblA][Table tblB]

**Appendix A. tblA:** Developed (substituted) alpha- and beta-carotene food composition table.

Food code^a^	Total carotene (*μ*g/100 g food portion)	Alpha-carotene (*μ*g/100 g food portion)	Beta-carotene (*μ*g/100 g food portion)	Method of substituition^b^	Food code^a^	Total carotene (*μ*g/100 g food portion)	Alpha-carotene (*μ*g/100 g food portion)	Beta-carotene (*μ*g/100 g food portion)	Method of substituition^b^
	
1-63	180	71	109	A	13-19b	400	34	366	A
1-64	200	79	121	A	13-19c	42	4	38	A
1-65	210	83	127	A	13-19d	36	3	33	A
1-66	160	63	97	A	13-19e	65	6	59	A
1-67	140	55	85	B	13-20a	160	14	146	A
1-68	100	39	61	A	13-24a	18	4	14	A
4-63a	180	71	109	A	13-24b	75	20	55	A
12-6a	480	328	152	A	13-26a	120	10	110	A
12-6b	500	337	163	A	13-26b	80	6	74	A
12-17a	620	565	55	A	13-26c	120	10	110	A
12-17b	520	474	46	A	13-27	320	15	305	A
12-35a	290	229	61	A	13-38a	19	11	8	A
12-35b	340	260	80	A	13-38b	26	16	10	A
12-36	8700	117	8583	A	13-42	85	51	34	B
12-38a	310	21	289	B	13-43	32	3	29	B
12-38b	320	22	298	B	13-46a	750	63	688	B
12-80a	2000	77	1923	A	13-49	12	1	11	B
12-80b	20000	769	19231	A	13-50	80	7	73	B
12-83a	44	22	22	A	13-51	90	8	83	B
12-83b	44	22	22	A	13-54	10	7	3	B
12-84a	60	30	30	A	13-55	13	9	4	B
12-84b	75	38	19	A	13-58	12	1	11	A
12-85	390	86	304	A	13-62	6	4	2	B
12-86c	480	97	383	A	13-63	1400	88	1313	A
12-86e	2000	46	1954	A	13-64	27	19	8	A
12-92a	250	123	127	A	13-65		577	263	A
12-92b	280	138	142	A	13-75	6	4	2	B
12-94a	7300	2263	5037	A	13-76	14	9	5	B
12-94b	8300	2590	5710	A	13-77	110	9	101	B
12-125a	400	9	391	A	13-80b	450	5	446	A
12-125b	330	6	266	A	13-88	11	3	8	A
13-1	120	9	25	A	13-90	9	2	7	A
13-11	12	3	9	B	16-19a	21000	1470	19530	A
13-17a	60	5	55	A	16-20	29000	7614	21386	A
13-17b	120	10	110	A	16-21a	13000	910	12090	A
13-18a	60	5	55	A	16-22a	13000	910	12090	A
13-18b	120	10	110	A	16-23a	14000	1400	12600	A
13-19a	75	6	69	A	16-25a	7000	598	6402	A

^a^ Food code used in the standard tables of food composition in Japan, the fourth revised edition^19^^)^.

^b^ See text for methods of substitution.

**Appendix B. tblB:** Foods of which carotene was only beta-carotene (alpha-carotene was not detected or negligible) by literature-based approach.

Method of substitution ^a^	Food code ^b^
A	2-5a, 2-5b, 2-5c, 2-6, 4-29, 7-7a, 7-7b, 7-8,7-11,7-12, 7-15a, 7-15b, 7-16, 7-17, 7-19, 7-27, 7-40a, 7-40b, 10-5a, 10-5b, 10-5c, 10-6, 10-7, 10-8, 10-9a, 10-9b, 10-10, 10-11, 10-14, 10-15, 10-16, 11-1, 11-2, 11-3a, 11-3b, 11-5b, 11-6a, 11-6b, 11-7a, 11-7b, 11-9a, 11-9b, 11-11a, 11-11b, 11-12, 11-14, 11-15, 11-16, 11-17b, 11-18, 11-19, 11-20, 11-22a, 11-22b, 11-22c, 11-22d, 11-22e, 11-22f, 11-22g, 11-22h, 11-22i, 11-23, 11-24, 11-25, 11-28, 11-29, 12-2a, 12-2b, 12-3a, 12-3b, 12-4a,12-4b, 12-8a, 12-8b, 12-9a, 12-9b, 12-10a,12-10b, 12-11, 12-14a, 12-14b, 12-15a, 12-15b, 12-15c, 12-15d,12-18a, 12-18b, 12-19a, 12-19b, 12-20a, 12-20b, 12-24a, 12-24b, 12-25a, 12-25b, 12-25c, 12-26a, 12-26b, 12-27a, 12-27b, 12-27c, 12-30a, 12-30b, 12-32a, 12-32b, 12-34a, 12-34b, 12-34c, 12-39a, 12-39b, 12-46, 12-48, 12-49a, 12-49b, 12-50, 12-53a, 12-53b, 12-54, 12-55a, 12-55b, 12-55c, 12-65, 12-66, 12-72, 12-73, 12-74a, 12-74b, 12-77a, 12-77b, 12-86a, 12-86b, 12-86d, 12-87a, 12-87b, 12-87c, 12-87d, 12-88, 12-89, 12-91a, 12-93a, 12-93b, 12-96, 12-97, 12-98a, 12-98b, 12-99, 12-101a, 12-101b, 12-101c, 12-104, 12-108a, 10-108b, 12-109a, 12-109b, 12-110a, 12-110b, 12-113a, 12-113b, 12-114a, 12-114b, 12-117a, 12-117b, 12-120, 12-121a, 12-121b, 12-122a, 12-122b, 12-123a, 12-123b, 12-137a, 12-137b, 12-137b, 12-138a, 12-138b, 12-139a, 12-139b, 12-140, 12-141, 13-2, 13-3, 13-4, 13-5, 13-6, 13-21a, 12-21b, 13-22, 13-23a, 13-25, 13-31, 13-32a, 13-32b, 13-45, 13-47, 13-48, 13-59a, 13-59b, 13-60, 13-61, 13-66a, 13-66b, 13-68, 13-69, 13-70, 13-71, 13-73, 13-79, 13-80a, 13-81a, 13-81b, 13-83a, 13-84a, 16-24a, 16-26a, 16-27a, 16-31, 17-6a, 17-6b, 17-6c
B	10-1, 10-2, 10-3, 10-4, 12-12a, 12-12b, 12-12c, 12-28a, 12-28b, 12-64a, 12-64b, 12-82a, 12-82b, 12-102a, 12-102b, 12-102c, 12-116a, 12-116b, 12-119a, 12-119b, 13-56, 13-78
C	4-7, 4-15, 4-43, 4-45, 4-46, 4-49, 4-50, 4-51, 4-52, 4-53, 4-67, 4-73a, 4-74, 8-88c, 12-13a, 12-13b, 12-76, 12-79a, 12-90, 12-132a, 12-132b, 12-132b, 12-133, 12-134a, 12-134b

^a^ See text for methods of substitution.

^b^ Food code used in the standard tables of food composition in Japan, the fourth revised edition.
